# Practice-oriented solutions integrating intraoperative electron irradiation and personalized proton therapy for recurrent or unresectable cancers: Proof of concept and potential for dual FLASH effect

**DOI:** 10.3389/fonc.2022.1037262

**Published:** 2022-11-14

**Authors:** Felipe A. Calvo, Adriana Ayestaran, Javier Serrano, Mauricio Cambeiro, Jacobo Palma, Rosa Meiriño, Miguel A. Morcillo, Fernando Lapuente, Luis Chiva, Borja Aguilar, Diego Azcona, Diego Pedrero, Javier Pascau, José Miguel Delgado, Javier Aristu, Alberto Alonso, Yolanda Prezado

**Affiliations:** ^1^ Department of Radiation Oncology, Clinica Universidad de Navarra, Madrid, Spain; ^2^ Medical Applications Unit, Centro de Investigaciones Energéticas, Medioambientales y Tecnológicas (CIEMAT), Madrid, Spain; ^3^ Department of Surgery, Clinica Universidad de Navarra, Madrid, Spain; ^4^ Department of Gynecology and Obstetrics, Clinica Universidad de Navarra, Madrid, Spain; ^5^ Department of Medical Physics, Clinica Universidad de Navarra, Madrid, Spain; ^6^ Department of Bioengineering and Aerospace Engineering, Universidad Carlos III de Madrid, Madrid, Spain; ^7^ Department of Radiology, Unit of Vascular Surgery and Interventional Radiology, Clinica Universidad de Navarra, Madrid, Spain; ^8^ Translational Research Department. Institut Curie, Université PSL, CNRS UMR, Inserm, Signalisation, Radiobiologie et Cancer, Orsay, France; ^9^ Université Paris-Saclay, CNRS UMR, Inserm, Signalisation, Radiobiologie et Cancer, Orsay, France

**Keywords:** electron FLASH, proton therapy FLASH, cancer, reirradiation, oligorrecurrent

## Abstract

**Background:**

Oligo-recurrent disease has a consolidated evidence of long-term surviving patients due to the use of intense local cancer therapy. The latter combines real-time surgical exploration/resection with high-energy electron beam single dose of irradiation. This results in a very precise radiation dose deposit, which is an essential element of contemporary multidisciplinary individualized oncology.

**Methods:**

Patient candidates to proton therapy were evaluated in Multidisciplinary Tumor Board to consider improved treatment options based on the institutional resources and expertise. Proton therapy was delivered by a synchrotron-based pencil beam scanning technology with energy levels from 70.2 to 228.7 MeV, whereas intraoperative electrons were generated in a miniaturized linear accelerator with dose rates ranging from 22 to 36 Gy/min (at Dmax) and energies from 6 to 12 MeV.

**Results:**

In a period of 24 months, 327 patients were treated with proton therapy: 218 were adults, 97 had recurrent cancer, and 54 required re-irradiation. The specific radiation modalities selected in five cases included an integral strategy to optimize the local disease management by the combination of surgery, intraoperative electron boost, and external pencil beam proton therapy as components of the radiotherapy management. Recurrent cancer was present in four cases (cervix, sarcoma, melanoma, and rectum), and one patient had a primary unresectable locally advanced pancreatic adenocarcinoma. In re-irradiated patients (cervix and rectum), a tentative radical total dose was achieved by integrating beams of electrons (ranging from 10- to 20-Gy single dose) and protons (30 to 54-Gy Relative Biological Effectiveness (RBE), in 10–25 fractions).

**Conclusions:**

Individual case solution strategies combining intraoperative electron radiation therapy and proton therapy for patients with oligo-recurrent or unresectable localized cancer are feasible. The potential of this combination can be clinically explored with electron and proton FLASH beams.

## Introduction

Precision oncology adapts an integral clinical vision to an individualized (personalized) care to patients with cancer (1, 2). Both cancer and patients are bio-heterogeneous, and their medical approach requires precise decisions selected on the basis of the bio-profile of “that cancer” and “that patient”. Precision oncology is a continuum of care across multiple specialties, creating a transverse reality requiring updated precision medicine in each interdisciplinary component of clinical practice, including diagnostic and therapeutic decisions (3). Transverse care incorporates a component of new radiobiology knowledge, state-of-the-art clinical and diagnostic techniques, and therapeutic innovation. Outside the committed team culture of the Multidisciplinary Tumor Board (MTB), evidence-based or innovated (evidence-generating) excellent clinical practice should be the goal of every oncology team. Precision oncology in up-to-date surgical, medical, and oncology care requires MTB enrichment (4). Local therapy for cancer control is an imperative requirement (*conditio sine qua non*) for disease-free and overall survival (5).

Radiotherapy (RT) is one of the most common and effective cancer treatments (6). Modern RT is recommended following personalized risk-adapted criteria in practice. In fact, 40% of patients who are cured of cancer will receive RT as part of their management, and around 50% of patients with cancer will require RT at some point during their treatment in high-income as well as low- and middle-income countries (7). In addition, it relieves symptoms in two of every three patients and, in general terms, is a crucial therapeutic component in three of every four patients with cancer. Furthermore, RT preserves organs and tissue structures and can be used in the context of radical treatment for oligometastatic and oligo-recurrent disease (6). Forecasts in healthcare systems in European countries suggested that, by 2025, indications for RT in all types of cancer will have increased by 5% to 35% across nations (8).

The clinical outcomes in RT have remarkably improved in the last decades, mainly due to technological advancements. Those have enabled dose reduction to the normal structures, thereby minimizing toxicity and facilitating dose escalation to the tumor, thus maximizing cancer control (9).

Among those developments, intraoperative electron radiation therapy (IOeRT) is a cancer treatment modality with a large body of evidence of successful use as risk adapted RT in multiple cancer sites, histological subtypes, and disease status (10). European Society for Radiotherapy and Oncology (ESTRO) guidelines are available in breast, rectal, and pancreas cancer and soft tissue sarcomas based on over 20,000 patients reported in the last decades (11–14).

Proton therapy is increasingly being used as a result of its inherent dosimetric advantages over photon therapy (15), which can result in less unnecessary irradiation of normal tissues (and presumably less toxicity) than traditional photon therapy (16). Proton therapy is proposed to have benefits for cancer of the central nervous system, head and neck, thorax, gastrointestinal tract, and breast, among others, although few direct comparisons of the two modalities have been made on the basis of clinical outcomes (17, 18).

FLASH irradiation is a novel RT technology using ultrahigh-dose rate (≥ 40 Gy/s) (19, 20). The irradiation times in FLASH-RT is 400-fold shorter than that in conventional RT (21). The FLASH effect (i.e., sparing of normal tissue) has been observed with both electron (19, 20, 22–26) and proton beams (27–31) in animal experiments. Tumor control has been observed to be equivalent to conventional RT, although the number of studies is limited (19, 29, 31, 32). In the first patient with T-cell cutaneous lymphoma who received FLASH-RT, the anti-tumor effect was rapid and long-lasting. Grade 1 epithelitis and edema was observed in the soft tissues surrounding the tumor (33). Considering that FLASH-RT can reduce the damage to healthy tissue and the advantages of the short treatment time, FLASH RT might become a paradigm change in RT technologies (34). Further research is needed to investigate the impact of the dose, fractionation and volume, oxygen content, and linear energy transfer on the FLASH effect.

In this work, we present a new personalized therapeutic strategy: intraoperative electron therapy combined with personalized proton therapy for recurrent or unresectable tumors and surgery. To the best of our knowledge, this is the first study of this kind. Furthermore, the availability of ultrahigh-dose rate clinical electron and proton devices (33, 35) is an opportunity to exploit the benefits of a potential dual FLASH effect.

## Materials and methods

### The models of recurrent or unresectable localized cancers for clinical practice innovation

In clinical practice, the availability of miniaturized electron linear accelerators, specifically designed for intraoperative electron irradiation and pencil beam proton beam therapy, offers many alternatives for dosimetric optimization in patients, requiring the surgical procedures for the radical treatment of their cancer (36).

A pilot experience based on individualized recommendation for rescue treatment in patients with recurrent or unresectable cancer is presented in this study. It includes a diversity of cancer sites, histology, and disease status.

The combination of components of RT delivering intraoperative electrons and external pencil beam proton therapy was considered an optimized strategy in terms of clinical benefit (improved therapeutic index, cancer control versus normal tissue toxicity) supported by the best dosimetric distribution in the target and preserving normal tissues from unnecessary irradiation. Surgical resection and surgical displacement of normal uninvolved mobile tissues and structures contributed further to improve the therapeutic index from combined modality therapy. It should be highlighted that, to the best of our knowledge, this is the first study of this kind.

These case solutions presented in this analysis are eloquent enough to show the potential of combining components of intraoperative electrons and external protons beams with FLASH characteristics achieving a final dose distribution with maximal protection of normal uninvolved tissues and an improved target selection (in the case of intraoperative electron beam, delivery is vision and surgically guided) (37).

## Results

### Dual electron-proton radiotherapy for semi-superficial recurrent cancer to prior surgery

Recurrent melanoma, head and neck, breast, and soft tissue sarcomas are cancer conditions that require re-resection and full component of RT to have an opportunity for local disease control. The post-resection tumor bed is at risk due to close or involved margins in the surgical specimen. A component of the dose can be delivered intraoperatively with electron beams protecting by displacement of normal uninvolved tissues (like the skin) (38–40) relevant for further surgical repairs maneuvers (tissue flaps reconstructions). The normal tissues exposed to the IOeRT component of the dose are muscle, soft tissues, ligaments, vessels, bone, and peripheral nerves. In experimental large animal models, the tolerance of the mentioned tissues and structures to IOeRT single high doses or single high doses combined with external normofrationated irradiation is favorable for 15 Gy. Peripheral nerves are sensitive to escalated doses. Above 20-Gy single dose or combined with external irradiation impaired motor function in extremities was described in 50% of animals (41, 42). The development of sensitive or motor neuropathy in patients with cancer is multifactorial and related to previous RT in the area, extended surgical resective procedures, or the evidence of previous pain, suggesting neural cancer involvement. In patients with asymptomatic peripheral nerves, the same tissue structures will be encompassed by the therapeutic region in proton therapy. Although flap reconstruction may improve tolerance to the external beam component of the RT dose, peripheral nerves will remain at risk for neuropathic damage adding the biological effect of protons and electrons. In this scenario, FLASH neuroprotective bioeffect could be exploited (43).

#### Example 1: Recurrent melanoma

**Figure 1:**
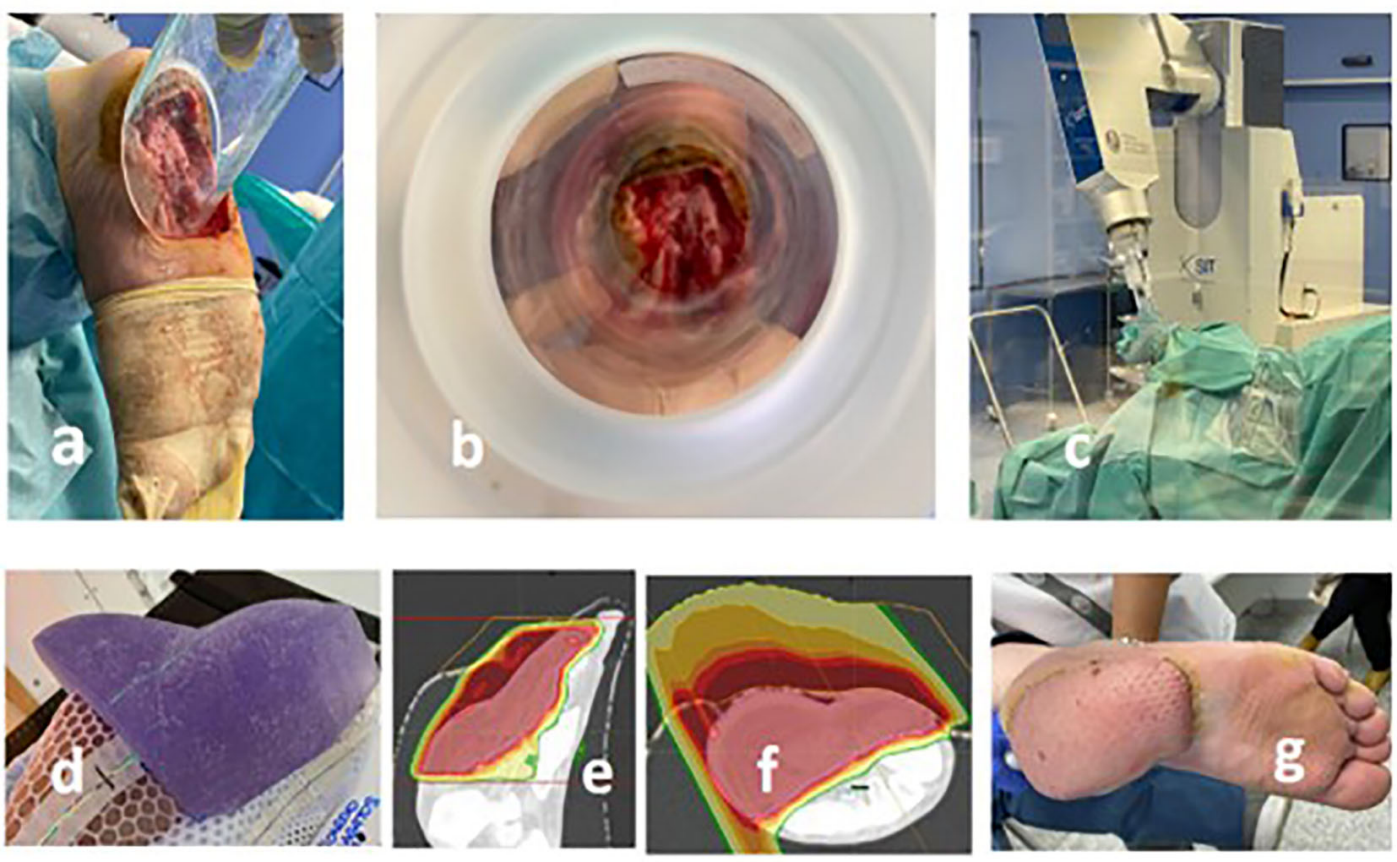
Recurrent plantar melanoma of the right foot treated with a combination of IOeRT and proton therapy. **(A)** Applicator positioning to encompass the post-resection bed including involved margins and the area at high risk for recurrence (skin is protected; key structure for the viability of the flap-repair maneuver). **(B)** Beam’s eye view of the target. **(C)** Linear accelerator and surgical room arrangement (notice the use of a beam-stopper). **(D)** Individualized bolus for proton therapy. **(E, F)** Dosimetric performance of the proton pencil beam. **(G)** Outcome of the myocutaneous flap employed for repair.

A 60-year-old man came to the outpatient clinic complaining of an ulcerative lesion on the right foot (plantar area) heel in April 2020. A biopsy was performed, which confirmed a histological diagnosis compatible with desmoplastic melanoma. Staging was completed with a PET–computed tomography (CT) that showed no other lesions. In May 2020, the tumor was resected with definitive diagnostic of desmoplastic melanoma (12 mm; Breslow, 1.4 mm; NLVI, NPNI), with positive deep margins. Multiple interventions were needed in June, July, and August, due to persistent evidence of positive margins. In September 2020, a new wide-excision resection was performed followed by IOeRT (20 Gy, 6-cm-diameter applicator and 12-MeV electron energy), and latissimus dorsi muscular flap reconstruction was assured. Proton therapy consolidation after an appropriate healing interval was planned and delivered in April 2021. He received a total dose of 30-Gy RBE (factor 1.1) in 10 fractions. Local edema and neurotoxicity grade 1 were the only reported side effects, without any additional relevant toxicity observed. To ensure an appropriate dose distribution in the tumor bed, an individualized silicon resin bolus with three-dimensional impression was used on the surface (**Figure 1**). Follow-up with clinical and physical exam and PET-CT was made every 3 months. In September 2021, the patient had a regional recurrence in a single inguinal node, which was biopsied in January 2022 with pathological confirmation of metastasis from melanoma. Because of this, in March 2022, he started a systemic therapy with immunotherapy (pembrolizumab). Currently, he had received two cycles with good tolerance and remains melanoma-free.

### Dual electron-proton radiotherapy in the management of intra-abdominal sarcoma recurrent to previous surgery

In patients with surgically resected sarcomas of the retroperitoneum randomized to 20-Gy intraoperative electron RT in combination with post-operative low-dose (35- to 40-Gy) external-beam RT or post-operative high-dose (50- to 55-Gy) external-beam RT alone, the number of locoregional recurrences was significantly lower among those who received intraoperative RT (6 of 15) than control patients (16 of 20).

Patients who received intraoperative RT had fewer complications of disabling radiation-related enteritis (2 of 15) than control patients (10 of 20), but radiation-related peripheral neuropathy was more frequent among those who received intraoperative RT (9 of 15) than among control patients (1 of 20) (44). This is an early firm evidence that 20-Gy single-dose IOeRT significantly contributes to neuropathy if combined with moderate external irradiation. At the same time, this is the first description of significantly improved therapeutic index on severe enteritis induced by surgery and high-dose external irradiation if a component of the dose is delivered intraoperatively (with small bowel displacement from the electron beam). In recurrent sarcomas, IOeRT as a component of treatment contributed to favorable outcomes: 5-year IORT in-field control, disease-free survival (DFS), and overall survival were 73%, 43%, and 52%, respectively (45). The external beam component of the RT dose will include small bowel and peripheral nerves. Proton therapy may optimize the contribution of unnecessary irradiation to the small bowel and using FLASH-proton technology to exploit the protective effect on intestinal structures and function described in animal models (46). In addition, the neuropathic damage from IOeRT might benefit from the biological neuroprotective effect of electron FLASH (43). Recurrent retroperitoneal sarcoma management with an IOeRT component is recommended in clinical practice by international guidelines (14).

#### Example 2: Recurrent soft tissue sarcoma

**Figure 2:**
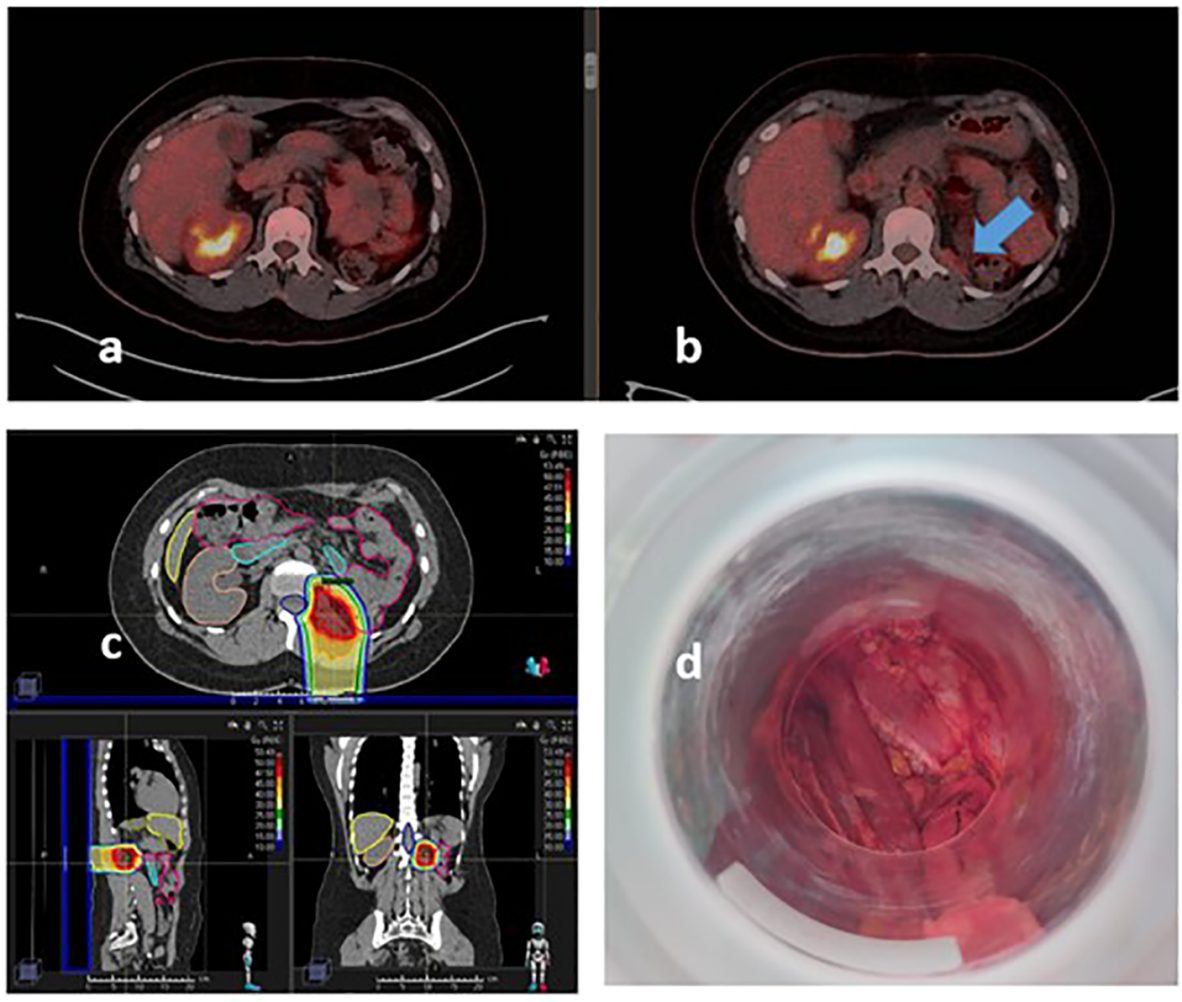
Recurrent synovial sarcoma of the lumbar fossae treated with proton therapy and IOeRT. **(A)** Restaging PET-CT after proton therapy. **(B)** Pre-proton therapy PET-CT showing metabolic active persistant disease before chemo-radiation in the retroperitoneal left lumbar fossae. **(C)** Single field proton beam arrangement and dose distribution. **(D)** Beam’s eye view of IOeRT: no mobile sensitive tissues in the target displaced mechanically.

A 40-year-old woman took in 2019 a fertility study with an abdominal echography and a CT scan, discovering an incidental lesion adjacent to the left kidney. The nephrectomy was made, but the primary lesion origin was the retroperitoneum. The pathology report was fusocelular sarcoma (15 cm larger dimension). A molecular panel showed a mutation without specific target therapy (FGFR3-R248C). Follow-up was recommended. On March 2021, a local recurrence was suggested by PET. In April, a resection (R1) follow by a hysterectomy and left oophorectomy was performed with pathological results of monofasic sinovial sarcoma G3 (60% necrosis; 12 cm; positive margin). In post-operative re-staging PET, residual tumor was observed nearby the staples in the surgical bed. With the diagnosis of persistent disease, the patient received a neoadjuvant chemotherapy (Adriamycin plus ifosfamide for three cycles between 16 August 2021 and 27 September 2021) followed by proton therapy between August and September 2021, reaching a total dose of 50-Gy RBE/20 fx (2.5 Gy/fx; factor, 1.1). Post-proton therapy restaging PET showed a complete metabolic response. Surgical exploration was recommended for dose escalation by IOeRT, whereas protection by displacement of small bowel, colon, and spleen was achieved (single dose of 12.5 Gy; 8-cm-diameter applicator, 45° beveled end; 12-MeV energy electrons), as well as surgical sampling of the tumor bed area (negative for residual sarcoma) (**Figure 2**).

### The potential of dual electron and proton radiotherapy in the management of recurrent rectal to previous surgery and radiotherapy

Multimodal strategies have been implemented for locally recurrent rectal cancer scheduled for surgical re-resection. Intraoperative electron irradiation (IOeRT) is a component of irradiation intensification that has been associated to long-term cancer control reported in patients who had multidisciplinary treatment (12). A particularly challenging group of patients with locally recurrent rectal cancer includes those who have received a course of pelvic irradiation. Re-irradiation is possible with some limitations regarding dose and volume. In general, re-irradiation targets are limited to the gross tumor volume with exclusion of the entire small bowel. Previously irradiated patients are at a higher risk of local relapse (37% *vs*. 22% at 3 years) due to proven biological adversity (radio resistance) and surgical limitations after previous resection (47). Nevertheless, studies have shown an overall improved oncological outcome in re-irradiated patients (48, 49). These results indicate the feasibility of re-irradiation containing an IOeRT component with electron beam energy technology. Nevertheless, a patient with three components of previous irradiation, including the tissues contained in the lateral pelvic wall and the dose-sensitive intrapelvic structures (bladder, intestine, and ureter), is a challenging scenario. The external beam component of the RT dose using proton therapy may optimize the contribution of unnecessary irradiation to the small bowel if a tissue spacer is fixed in the homolateral hemipelvis during the re-resection plus IOeRT procedure. Proton therapy dosimetry is accurate enough to avoid contribution to the intrapelvic structures due to this maneuver. From FLASH-proton technology protective effect on intestinal structures and function described in animal models (46), we could expect an additional advantage. Previous neuropathic damage from hypofractionated techniques (high-dose rate brachytherapy and stereotactic irradiation) is expected in this scenario, and IOeRT will further increase this risk. The largest clinical experience reported using IOeRT includes 50 patients evaluable for neurotoxicity analysis. Of the 50 patients evaluable for neurotoxicity analysis, 16 (32%) developed peripheral neuropathy consisting of pain in 16 patients, numbness and tingling in 11, and weakness in 8. The pain, numbness, and tingling were resolved in about 40% of patients, whereas weakness was resolved in only (12.5%). The development of neurotoxicity was more common at IORT doses of 1,500 cGy or more versus 1,000 cGy (50). The neuroprotective effect of electron FLASH in re-irradiated peripheral nerves is a model to be tested with a relevant clinical potential in radiation oncology practice (43).

#### Example 3: Recurrent rectal cancer

**Figure 3:**
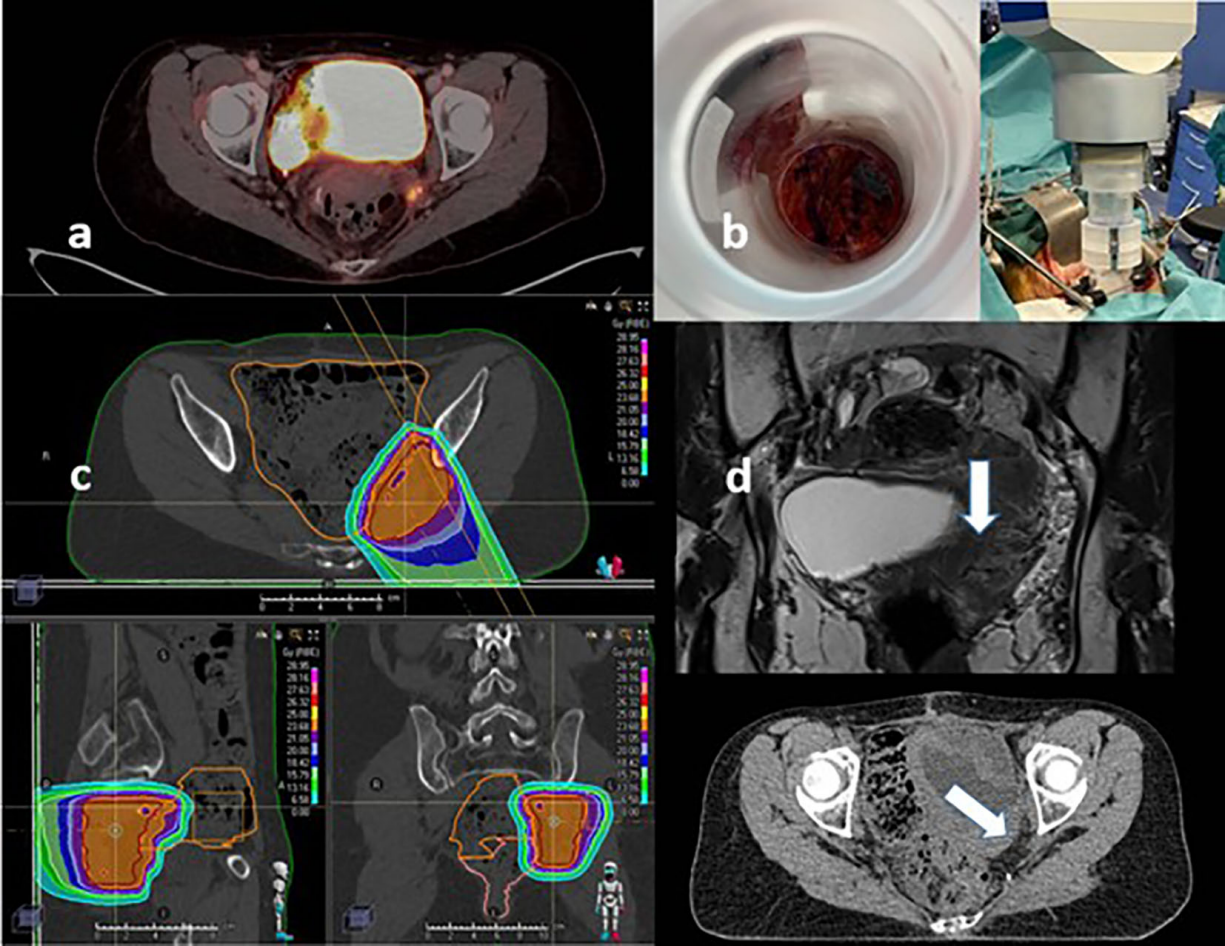
Oligo-recurrent rectal cancer treated with proton therapy and IOeRT under re-irradiation conditions (previous treatment components included neoadjuvant chemoradiation, hypofractionated perioperative high-dose rate brachytherapy, and stereotactic body radiotherapy). **(A)** Oligo-recurrent pelvic disease for small bowel displacement progressing after previous tri-irradiation from the proton beam. **(B)** Beam’s eye view of the target and post-docking view of the surgical area. **(C)** Proton beam field arrangement and dosimetric distribution. **(D)** Spacer made of epiplon at the time of surgical resection and IOeRT procedure.

A 52-year-old woman was diagnosed, in December 2015, of a locally advanced colorectal cancer (cT3 cN+). She received preoperative chemoradiation (long course: 50 Gy/20 fx with concurrent 5-FU) followed by surgery (left hemicolectomy and lymphadenectomy). The final anatomopathological results confirmed complete pathological response (ypT0 pN0) in the surgical specimen. Later, she completed adjuvant chemotherapy (raltitrexed and oxaliplatin for six cycles) and then follow-up. In 2018, because of rectal bleeding and increase in tumor marker CEA (6.1 ng/ml), an echoendoscope found of a local recurrence located 5 mm from anal canal. A low-anterior resection was performed (20 November 2018), and, because of close margins status (<1 mm), a multicatheter high-dose rate brachytherapy procedure was added (24 Gy in 3 days with a total of 6 fractions, bid, 4–6 h). In November 2019 restaging PET-CT described a suspicious new local recurrence in left pelvic wall marginal to the brachytherapy target volume. The patient received stereotactic body RT (SBRT) (45 Gy in 15 fractions) and chemotherapy as systemic treatment with irinotecan and bevacizumab (for six cycles, last in March 2020). In May 2021, oligometastatic progression was established (one lung metastasis of 8 mm) and new local recurrence (pelvic implant in the remaining mesorectum). The rescue recommendation was atypical segmentectomy of LSI VATS (June 2021), plus salvage surgery of pelvic implants in July 2021 boosted by IOeRT (15 Gy; 12.5 MeV; 7-cm-diameter applicator, 30° beveled end) and proton therapy after using an epiplon flap over the pelvic wall to displace and protect the small bowel as the limiting tissue at risk (a total dose of 25 Gy in 10 fractions was given, 2.5 Gy/fraction RBE with factor of 1.1) (**Figure 3**).

### The potential of dual electron and proton radiotherapy in the management cervix cancer initially treated with radical radiotherapy and rescue for a regional recurrence with risk of overlapping radiation volumes

The feasibility and long-term outcome of surgery combined with intraoperative electron radiotherapy (IOeRT) as rescue treatment in patients with recurrent and/or metastatic oligotopic extrapelvic cancer has reported that 86% of patients experienced local recurrence and 53.5% developed distant metastasis. Overall survival at 2 and 5 years was 57% and 35%, respectively. Local recurrence was significantly affected by microscopic cancer present in more than 50% of specimen fragments (38% *vs*. 9%, p = 0.02) (51). A subgroup of patients with para-aortic lymph-node oligometastases from gynecological malignancies treated with an IOeRT containing multimodal protocol obtained the following outcomes: with a median follow-up time of 55 months; 5-year loco-regional control; DFD; and overall survival rates were 79%, 44%, and 49%, respectively. On multivariate analysis, there is no EBRT treatment to the para-aortic region (p = 0.03), and time interval from primary tumor diagnosis to relapse <24 months (p = 0.04) remained significantly associated with locoregional recurrence. On multivariate analysis, only R1 margin status (p = 0.01) was significantly associated with overall survival (52).

The dosimetric comparison between pencil beam scanning proton therapy and intensity-modulated radiation therapy for pelvic and para-aortic lymph node disease in gynecologic carcer and acute toxicities associated with extended-field Pencil-Beam Scanning (PBS) has been analyzed. The organs at risk considered included pelvic bone marrow, small bowel, large bowel, rectum, bladder, and kidneys. Proton therapy significantly reduced small bowel dose volumes from 0 to 27.5 Gy, large bowel dose volumes from 0 to 31.6 Gy, bladder dose volumes from 0 to 27.3 Gy, and rectal dose volumes from 0 to 7.6 Gy (all P <.05) (53).

The tissues and structures that are exposed to the escalated doses of intraoperative electron irradiation are large vessels, soft tissues, muscle, prevertebral ligament, and superior portion of the vertebral body (bone). In animal experiments, high single doses of intraoperatively delivered electron beam irradiation have considered all this tissues relatively radioresistant (minor signs of histological or radiological changes with doses up to 20-Gy single fraction) (44, 54, 55). This level of tolerance includes arteries and veins with previous surgical manipulation for grafts (56). A cohort of 195 long-term surviving patients (over 5 years of follow-up) after IOeRT containing multimodal treatment vertebral collapse (14%), vascular damage (14%), and grade 3 soft tissue fibrosis (7%) has been described (57). Post-lymphadenectomy tissues exposed to IOeRT have a level of loss of capillary vascularization that influences the radiation response of these tissues.

In a post-lymphadenectomy scenario for the rescue of para-aortic nodal recurrences, the external beam component of the RT using proton therapy is optimized by the dosimetric behavior of the proton beam, limiting the contribution of unnecessary irradiation to the small bowel, both in the upper abdominal region and in the pelvic region in an area at high risk for overlapping with the previous external and brachytherapy treatments. IOeRT is particularly protective in the para-aortic region of small and large bowel, kidneys, and ureters by mechanical displacement of these structures and organs. The possibility of using FLASH rate delivery in both elements of radiation treatment (IOeRT and proton therapy) might significantly contribute to improved radiobiological protection of normal tissues at risk (43, 46).

#### Example 4: Recurrent cervix cancer

**Figure 4:**
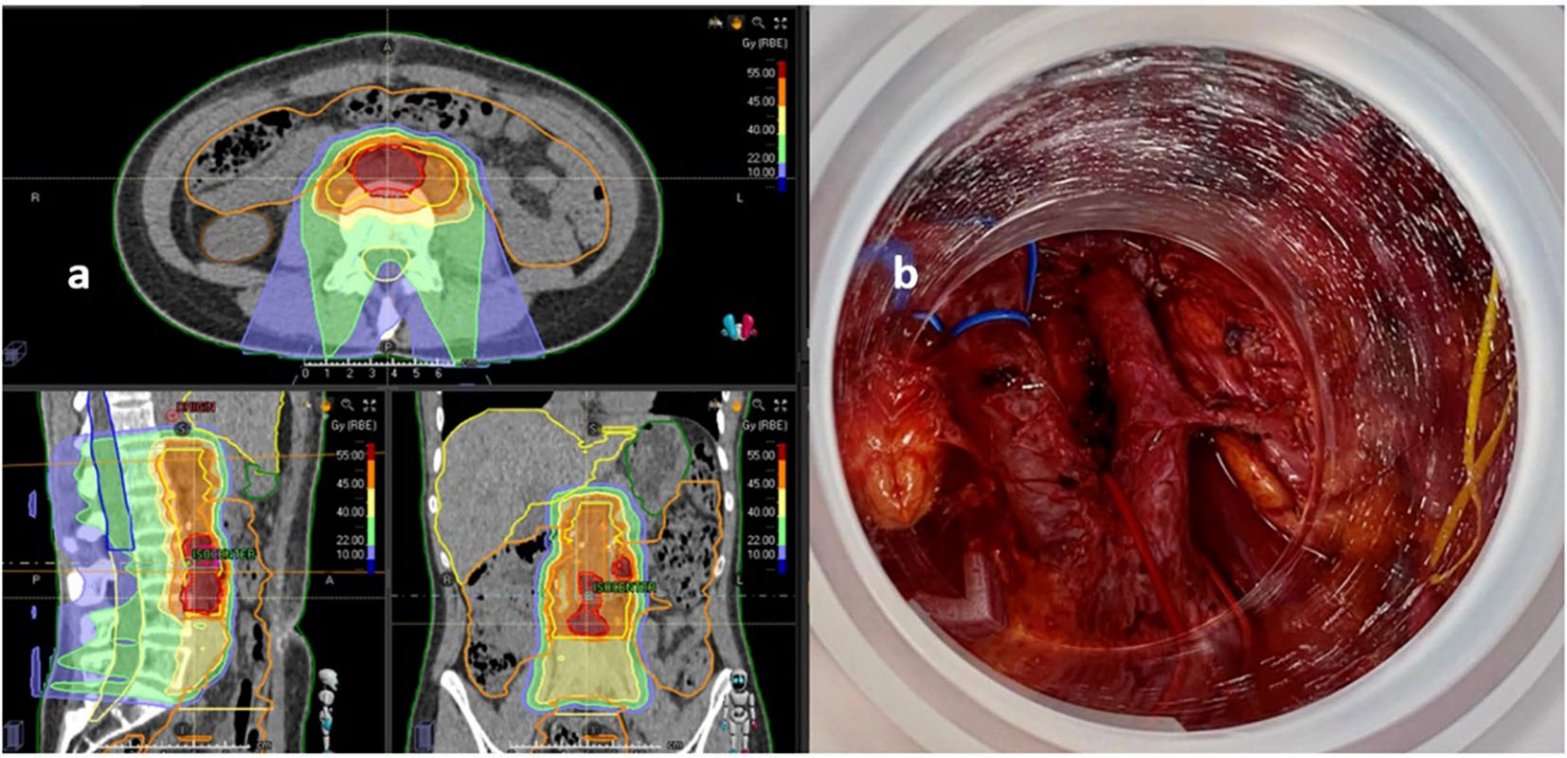
Recurrent cervix cancer patient treated with lymphadenectomy, IOeRT, and proton therapy after nodal oligo-progression outside the previous areas of external irradiation and brachytherapy. **(A)** Proton therapy field arrangements and dose-distributions. **(B)** Beam’s eye view of the IOeRT target (notice the absence of dose-sensitive tissues such as bowel and ureters).

A 34-year-old woman, because of vaginal bleeding, was diagnosed by conization with a cervical adenocarcinoma stage IB (>5-mm-depth stromal invasion; 9 mm in greatest dimension). She was treated with radical trachelectomy and pelvic nodal dissection without further residual tumor found. In 2019, the patient developed pelvic pain and multiple studies including cytology, pelvic MRI, and PET-CT, confirming the presence of a local recurrence in the right parametrial region. She received concurrent chemo-RT with cisplatin (40 mg/m^2^ weekly). The pelvic irradiation consisted in 50 Gy to macroscopic tumor recurrence and 45 Gy to nodal pelvic region, in 20 fractions both. Afterward, high-dose rate brachytherapy was delivered using intracavitary application first (Fletcher system; two sessions of 7.39 Gy prescribed to D90 CTV-HR) and, later, a second interstitial application (Utrech system; two sessions of 7.39 Gy prescribed to D90 CTV-HR). In the oncological follow-up, a complete clinical and radiological response was observed for 14 months. Thirty-five months later, pelvic MRI and PET-CT revealed a locoregional relapse located in the para-aortic and inter-aorto-cava region, compatible with nodal metastases. Rescue lymphadenectomy was associated to IOeRT boost (15 Gy; 12.5 MeV; 70-cm-diameter applicator, 30° beveled end). External pelvic irradiation was delivered with proton therapy (54 Gy to CTV-HR at high risk; 45 Gy to CTV-N nodal elective irradiation in 25 fractions) (**Figure 4**). Afterward, the patient received additional systemic treatment with Cemiplimab based on the EMPOWER study results and PD-L1 expression (TPS 14%).

### The potential of dual electron and proton radiotherapy in the management of locally advanced unresectable pancreatic cancer

Clinical results of IOeRT used as a boost strategy (integrated for a dose escalation multimodality approach) or as the only RT component were tested for localized unresected, borderline, or post-resection pancreatic cancer, and international guidelines are available (11, 12). Unresectable disease categories benefit from dose-escalated chemoradiation strategies in the context of active systemic therapy and potential radical surgery (58). Prolonged preoperative treatment may act as a filter for selecting patients with occult resistant metastatic disease (59). Long-term survivors were observed among unresected patients treated with external beam RT and an IOeRT boost (OS 6% at 3 years; 3% >5 years) (60, 61). Detailed autopsy analyses of the radiation effects on the pancreas and adjacent tissues have been reported. In unresectable pancreatic carcinoma, the major expression of intraoperative irradiation with external beam irradiation is a progressive fibrosis of the pancreas with vascular sclerosis, nerve degeneration, atrophy of acinar cells, and atypical changes in the ducts of the pancreas, as well as degenerative changes of the pancreatic tumor (62). Histological changes related to radiation were generally manifested as fibrosis. In addition, mild fibrotic changes in retroperitoneal soft tissues and mild hypocellularity in vertebral bone marrow were consistently present in patients treated for pancreatic carcinoma. Fibrosis of the soft tissues of the porta hepatis without narrowing of the bile duct was also present together with perineural fibrosis. Significant radiation-related changes were generally not observed in major blood vessels, intestine, or ureter. Intact irradiated primary tumors consistently displayed necrosis (63). Several animal experiments have explored tolerance and pathologic changes associated to escalated doses of electron irradiation in intestine and bile duct (64), pancreas (65), and duodenum (66). Duodenitis has been described as a side effect of IOeRT in clinical studies (67, 68). Animal experiments have diminished the duodenal toxicity using with intraluminal WR2721 (69).

On the other hand, proton therapy dosimetry may be advantageous in the case of unresectable pancreatic cancer with potential protection of normal adjacent tissues from unnecessary low and intermediate (occasional high doses) of irradiation at the kidneys, transverse colon, small bowel, stomach, spleen, liver, bile duct, large vessels, vertebral bodies, spinal cord, and duodenum (70). Surgical spacer placement for subsequent proton RT can improve the dose intensity covering 95%, mean, and minimum doses for the gross tumor volume, as well as the clinical and planning target volume based on the Dose-Volume Histogram (DVH), while respecting the dose constraints of the gastrointestinal tract. The effects of the spacer in clinical terms clinical described were 22% gastrointestinal ulcer (grade 2) and 11% gastric perforation (grade 4) (71).

The possibility of using FLASH rate delivery in both elements of radiation treatment (IOeRT and proton therapy) might significantly contribute to improved radiobiological protection of a large proportion of upper abdominal normal tissues at risk including gastrointestinal structures and nerves (43, 46). This scenario can be exploited in dose escalation strategies to further improve local control and long-term survivors.

#### Example 5: Localized unresectable pancreatic cancer

A 70-year-old man is complaining of abdominal pain. Angio CT showed (24.08.2021) a 3 × 3 cm mass in the body of the pancreas with a 360° encasement of the superior mesenteric artery and 90° the coeliac trunk. Minor dilatation of the distal coledocum was observed. PET-CT described a pancreatic mass with SUVmax of 9.8 and suspicious nodal retropancreatic disease (SUVmax of 3.6; CA 19.9; 215.8 U/ml). Staging laparoscopy was negative for peritoneal extension.

**Figure 5:**
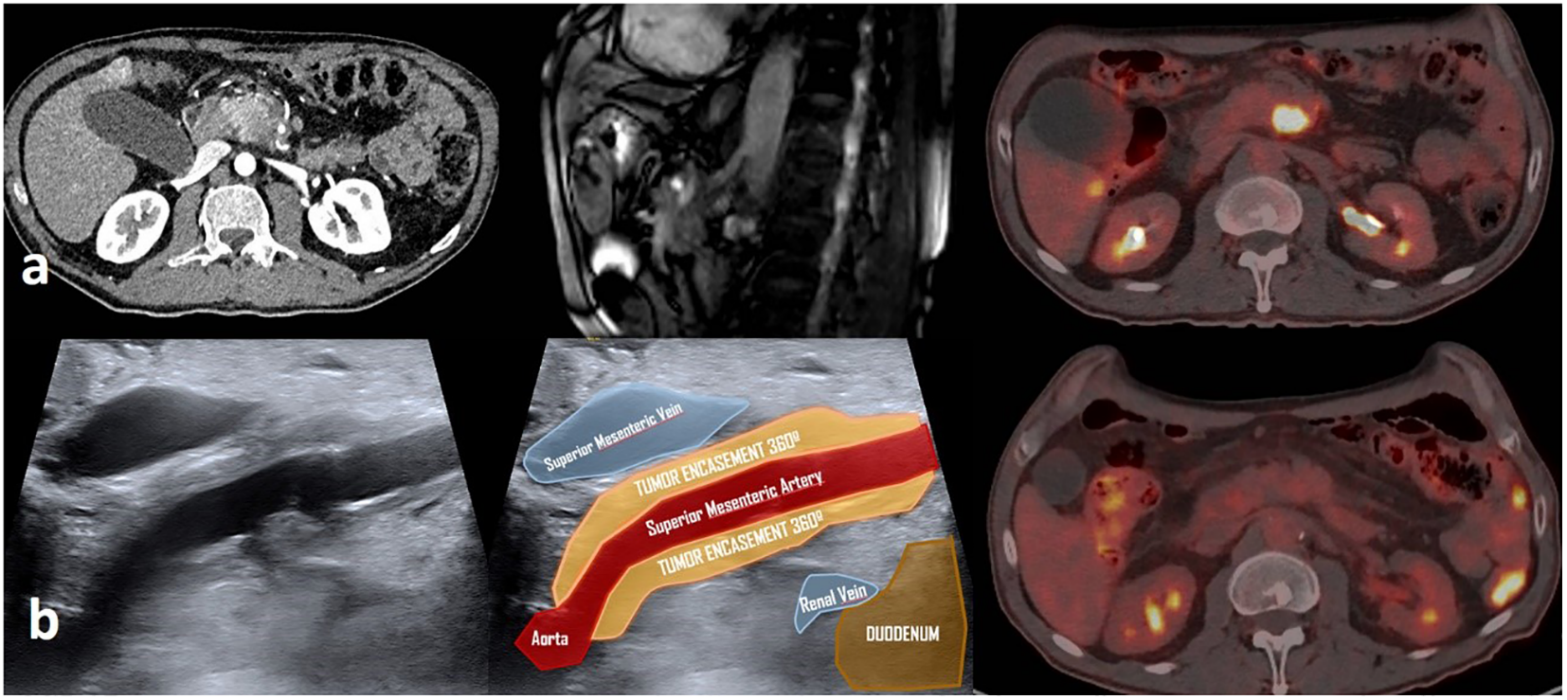
Imaging studies evolution in a patient with unresectable pancreatic cancer treated with induction chemotherapy FOLFIRINOX, neoadjuvant chemo-radiation with proton therapy and IOeRT boost consolidation. **(A)** Initial diagnostic studies (angioCT, MRI, and PET-CT) showing the vascular involvement and unresectable nature of the disease. **(B)** Post-treatment imaging studies showing a complete metabolic remission and a 360° encasement of the superior mesenteric artery.

**Figure 6:**
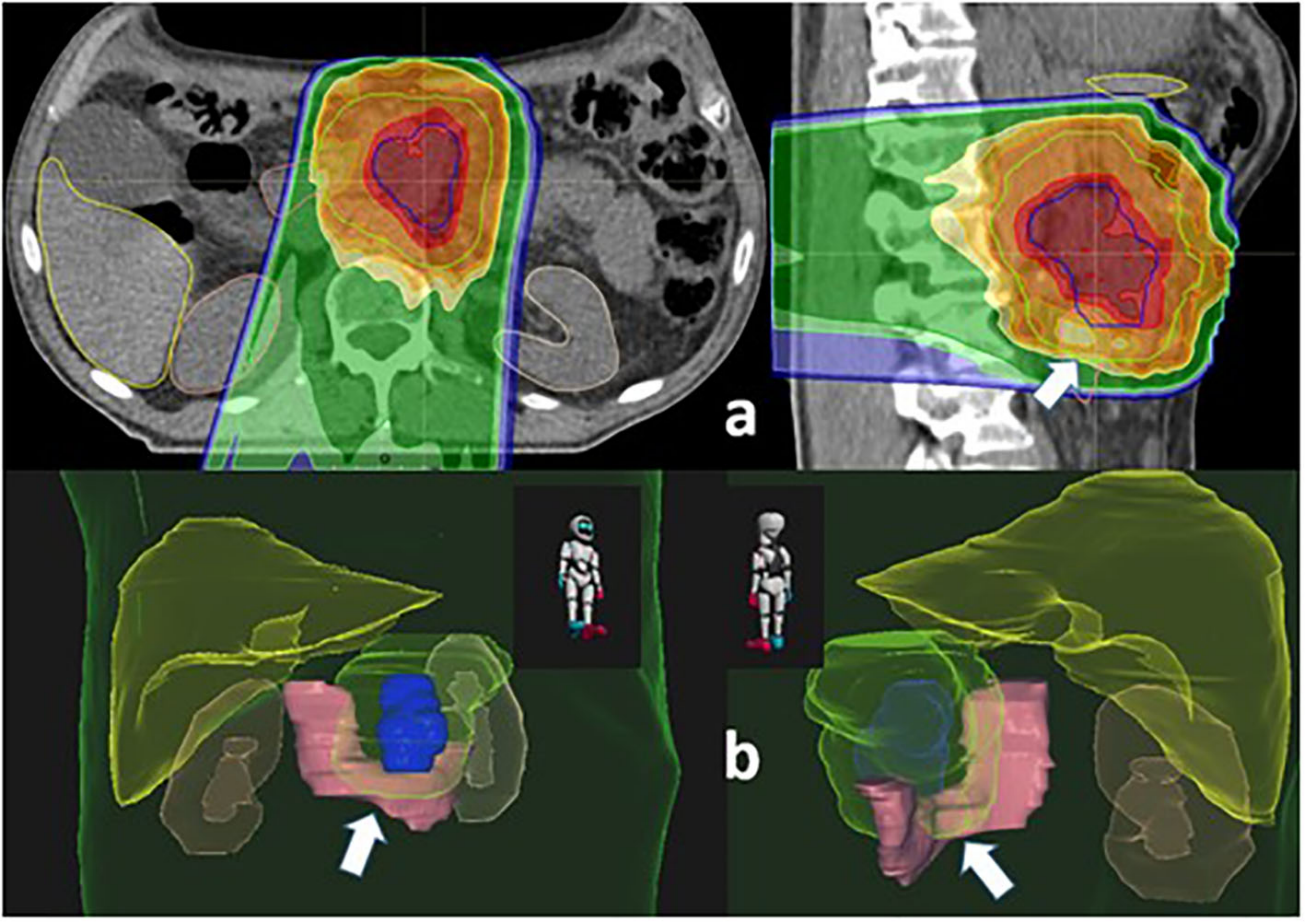
Treatment planning images from proton therapy. **(A)** Two-dimensional representation (red, 54 Gy; green line, 45 Gy). **(B)** Three-dimensioanl reconstruction dose distribution with special emphasis of the CTV and the duodenum (solid pink structure).

Neoadjuvant treatment consisted in four cycles of FOLFOXIRI. Response assessment was favorable: abdominal pain has improved, and marker was normalized. Restaging studies showed complete metabolic response, minor decrease in size of the pancreatic mass, and persistent circumferential involvement of the superior mesenteric artery (**Figure 5**). For Multi-Field Optimization (MFO) from 23 November 2021 to 23 December 2021, PBS proton therapy was delivered to a Clinical Target Volume (CTV), encompassing all the involved area determined by a fusion of PET-CT and MRI images. Dose per fraction was 2.3 Gy (RBE 1.1), and the total dose was 45 Gy in the elective nodal region and 54 Gy in the macroscopic metabolic active initial disease (integrated boost; dose per fraction, 2.7 Gy) (**Figure 6**). Re-restaging PET-CT was negative in the upper abdominal region.

**Figure 7:**
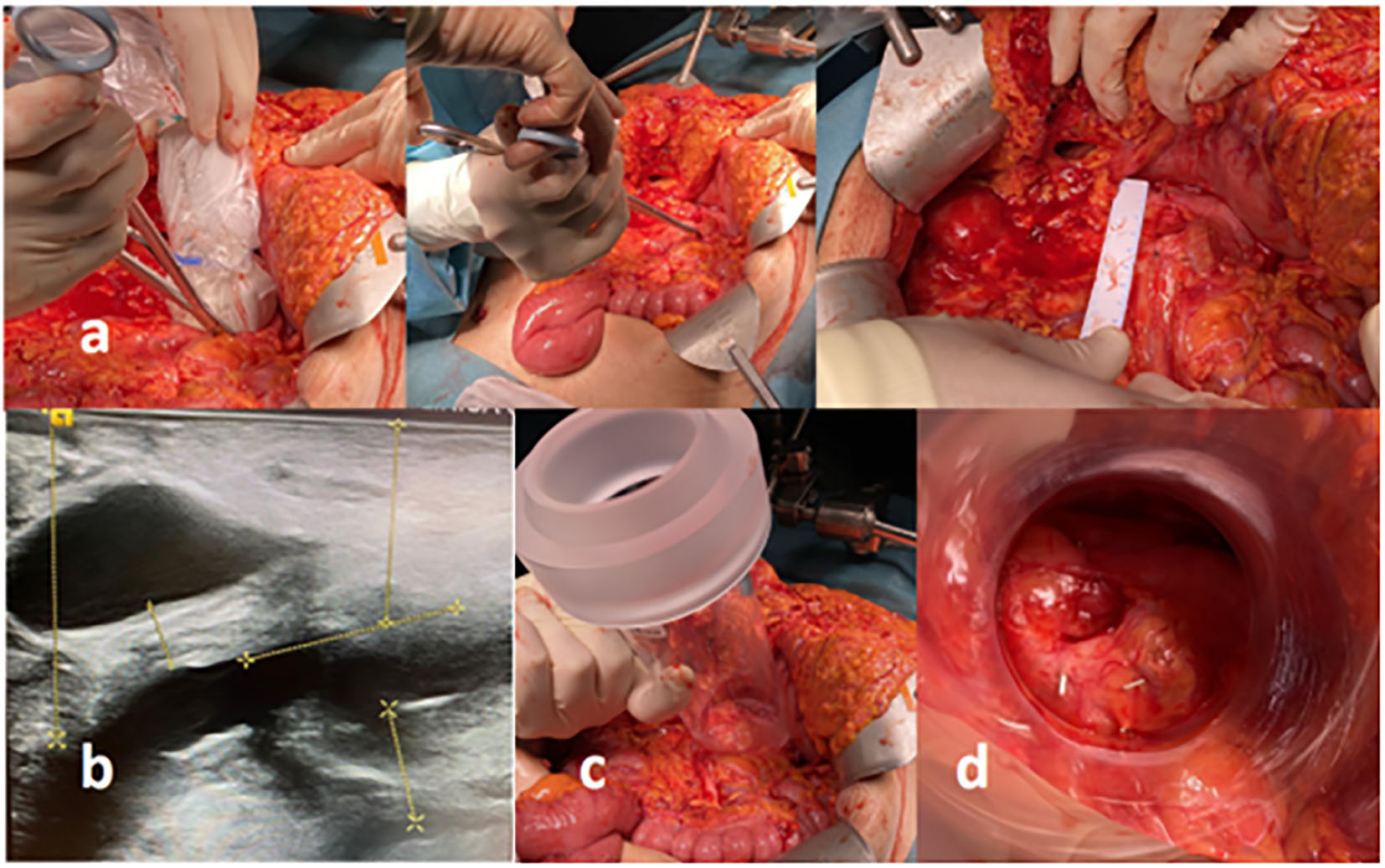
Combined surgical and IOeRT procedure in a patient with unresectable cancer of the body of the pancreas with 360° involvement of the superior mesenteric artery explored and treated after intense neoadjuvant therapy and complete metabolic remission (including tumor markers normalization). **(A)** Surgical maneuvers to guide the IOeRT procedure (ultrasound delimitation of residual abnormalities; target definition with surgical clips; measurements for applicator selection. **(B)** Ultrasound measures for electron energy, applicator size selection, and the effect of the displacement of the duodenum from the target by the use of a spacer. **(C)** External view of the surgical field and IOeRT applicator positioning. **(D)** Beam’s eye view of the target (notice the margin around the markers fixed at the time of intraoperative ultrasound assessment).

Subsequently, laparotomy and tumor exposure to intraoperative electron irradiation were preformed delivering 15 Gy of 12-MeV electrons using a 6-cm-diameter applicator, 30° beveled end (**Figure 7**). Displaced and protected from the electron beam were the stomach, transverse colon, small bowel, liver, and duodenum (transient displacement with mechanical spacer). Patient was discharged from the hospital in day 3, post-operative period.

## Discussion

The results of IOeRT in the treatment of oligo-recurrent or unresectable cancer in the abdomino-pelvic regions have reported long-term controlled patients in colorectal cancer (72), gastric cancer (73), gynecologic cancer (74), sarcomas (45), and kidney cancer (75). The evidence of survivors after components of high precision RT with or without surgery (IOeRT and stereotactic body irradiation) has promoted a comprehensive system for characterization and classification of oligocancer disease behavior by the European Society for Radiotherapy and Oncology and European Organization for Research and Treatment of Cancer developing the OligoCare project (76). An interesting subclassification (oligorecurrence, oligoprogression, and oligopersistence), considering whether oligometastatic disease is diagnosed during a treatment-free interval or during active systemic therapy and whether an oligometastatic lesion is progressing on current imaging is proposed to further understand clinical results observed. Local interventions are also relevant in the context of the addition of systemic therapy to enhance the local control promotion effect to prolong the systemic therapy-free interval. The contribution of proton therapy to control locally advanced cancer requiring chemoradiation is being built-up under the basis of exploiting the equation, in which dosimetric benefit is the rational argument to achieve a clinical benefit expectation (77).

To the best of our knowledge this is the first study on personalized treatments combining IOeRT and proton therapy in recurrent tumors. This investigation in five patients shows a significant increase in the early therapeutic index and illustrates the potential of this dual RT combination. The outcome might be further enhanced by using new dose delivery methods, such as FLASH-RT.

The FLASH effect has been observed both with electrons (19, 20, 22, 23) and proton beams (27–31). Should the FLASH effect be confirmed in temporal fractionation schemes, the therapeutic strategy presented in this study would benefit from an enhanced normal tissue sparing coming from this potential double FLASH effect (electron and proton FLASH-RT).

To date, the biological mechanisms under FLASH-RT remain elusive. Several mechanisms have been proposed in the literature, namely, the lower creation of oxygen reactive species depending on tissue hypoxia level or a different immune activation through the systemic immune cells or inflammatory response in the tissue (20, 24, 78–81). However, there is currently a lack of substantial biological data to support this hypothesis, and, for the moment, only few evaluations were performed. The published data show an increase in CD8^+^ cell infiltration (79, 82); however, this increase has been observed to be higher than that in conventional RT only in subcutaneous models (79) in contrast with the more realistic orthotopic models employed by Eggold et al. (82).

In any case, FLASH-RT might offer an advantage to be combined with immunotherapy as the high doses per fraction needed to mount an effective immune response are more likely to better tolerance than in conventional regimes. Along this line, the double irradiation electron and proton FLASH-RT, with a natural time delay of some weeks between them, could further enhance the immune response as observed in PULSAR irradiations (83). Moreover, a recent work of Tinganelli et al. (84) indicated a decrease in lung metastasis following FLASH with carbon ions, further suggesting a different immune modulation in FLASH radiations, which achieves abscopal effect and needs to be addressed in future works.

To move toward FLASH-RT clinical trials of large radioresistant or recurrent tumors (the ones that would most benefit from the normal tissue preservation of FLASH therapy), further and comprehensive studies exploring FLASH-RT are still lacking. First, the current evidence on tumor control by FLASH therapy is much more limited than the normal tissue sparing, and most of the studies use a single dose scheme. Studies assessing the maximum dose and the time delay between fractions for which the FLASH effect still stands are requested. All those evaluations are necessary to critically evaluate its toxicity and efficacy under a more clinically relevant scenario. Dose–volume effects can also have an important impact on FLASH-RT (85). Furthermore, oxygen content can be crucial in the appearance of FLASH effect (24). No evaluation of the FLASH effect in re-irradiations has been carried out. The radiobiological mechanisms governing the differential dose sparing in FLASH irradiations and their efficacy or even their probability of occurrence are still unclear. To analyze these uncertainties, FLASH treatments require additional standardized reporting of the application and of the physical and technical parameters that may influence the efficacy of the FLASH effect as argued in the previous review (86).

Despite those many open questions, the therapeutic strategy proposed in this work has the potential to maximize the benefits of FLASH-RT due to the double irradiation with both electrons and protons. The already existing availability of clinical devices (both with electrons and protons) able to deliver the ultrahigh-dose rates and beam characteristics needed to trigger the FLASH effect is an opportunity to expedite translation of this technology into pragmatic clinical trials.

## Conclusions

The treatment of recurrent and radiation-resistant tumors is still restricted by the dose-limiting normal tissue complications rates. The first clinical combination of electron IORT and personalized proton therapy presented in this work has led to a significant improvement of the early treatment outcome in a series of particularly complex patients with oligo-recurrent or unresectable localized cancer. Moreover, recent technological advances have enabled the use of electron and proton beams at ultrahigh-dose rates. Therefore, this combination is a differential and promising model to investigate a double FLASH effect contribution on normal tissues tolerance and dose escalation strategies.

## Data availability statement

The original contributions presented in the study are included in the article/supplementary material. Further inquiries can be directed to the corresponding author.

## Ethics statement

The studies involving human participants were reviewed and approved by Ethics Committee of Clinica Universidad de Navarra. The patients/participants provided their written informed consent to participate in this study. Written informed consent was obtained from the individual(s) for the publication of any potentially identifiable images or data included in this article.

## Author contributions

Conceptualization: FC, JA, MC and YP. Data curation: FC, AA, JS, MC, JA, RM, JPas, FL, BA, LC, MM, JP, JD, AAlon, and YP. Formal analysis: FC, JS, and JP. Investigation: FC, AA, MM, RM, BA, DP, DA, AAlon, and YP. Methodology: JS, AA, DP, DA, and YP. Project administration: FC. Supervision: FC, JA, and AA. Writing-original draft: FC, AA, JS, and YP. Writing-review and editing: FC, YP, JA, JS, RM, JP, JPas, LC, DP, DA, AAlon, and AA. All authors contributed to the article and approved the submitted version..
