# Corrigendum: Practice-oriented solutions integrating intraoperative electron irradiation and personalized proton therapy for recurrent or unresectable cancers: Proof of concept and potential for dual FLASH effect

**DOI:** 10.3389/fonc.2022.1116433

**Published:** 2023-01-18

**Authors:** Felipe A. Calvo, Adriana Ayestaran, Javier Serrano, Mauricio Cambeiro, Jacobo Palma, Rosa Meiriño, Miguel A. Morcillo, Fernando Lapuente, Luis Chiva, Borja Aguilar, Diego Azcona, Diego Pedrero, Javier Pascau, José Miguel Delgado, Javier Aristu, Alberto Alonso, Yolanda Prezado

**Affiliations:** ^1^ Department of Radiation Oncology, Clinica Universidad de Navarra, Madrid, Spain; ^2^ Medical Applications Unit, Centro de Investigaciones Energéticas, Medioambientales y Tecnológicas (CIEMAT), Madrid, Spain; ^3^ Department of Surgery, Clinica Universidad de Navarra, Madrid, Spain; ^4^ Department of Gynecology and Obstetrics, Clinica Universidad de Navarra, Madrid, Spain; ^5^ Department of Medical Physics, Clinica Universidad de Navarra, Madrid, Spain; ^6^ Department of Bioengineering and Aerospace Engineering, Universidad Carlos III de Madrid, Madrid, Spain; ^7^ Department of Radiology, Unit of Vascular Surgery and Interventional Radiology, Clinica Universidad de Navarra, Madrid, Spain; ^8^ Translational Research Department, Institut Curie, Université PSL, CNRS UMR, Inserm, Signalisation, Radiobiologie et Cancer, Orsay, France; ^9^ Université Paris-Saclay, CNRS UMR, Inserm, Signalisation, Radiobiologie et Cancer, Orsay, France

**Keywords:** electron FLASH, proton therapy FLASH, cancer, reirradiation, oligorrecurrent

**Error in Author List**

In the published article, there was an error in the author list; author Alberto Alonso was erroneously excluded. The corrected author list appears below.

Felipe A. Calvo^1*^, Adriana Ayestaran^1^, Javier Serrano^1^, Mauricio Cambeiro^1^, Jacobo Palma^1^, Rosa Meiriño^1^, Miguel A. Morcillo^2^, Fernando Lapuente^3^, Luis Chiva^4^, Borja Aguilar^5^, Diego Azcona^5^, Diego Pedrero^5^, Javier Pascau^6^, José Miguel Delgado^1^, Javier Aristu^1^, Alberto Alonso^7^ and Yolanda Prezado^8,9^

^1^ *Department of Radiation Oncology, Clinica Universidad de Navarra, Madrid, Spain*

^2^ *Medical Applications Unit, Centro de Investigaciones Energéticas, Medioambientales y Tecnológicas (CIEMAT), Madrid, Spain*

^3^ *Department of Surgery, Clinica Universidad de Navarra, Madrid, Spain*

^4^ *Department of Gynecology and Obstetrics, Clinica Universidad de Navarra, Madrid, Spain*

^5^ *Department of Medical Physics, Clinica Universidad de Navarra, Madrid, Spain*

^6^ *Department of Bioengineering and Aerospace Engineering, Universidad Carlos III de Madrid, Madrid, Spain*

^7^ *Department of Radiology, Unit of Vascular Surgery and Interventional Radiology, Clinica Universidad de Navarra, Madrid, Spain*

^8^ *Translational Research Department. Institut Curie, Université PSL, CNRS UMR, Inserm, Signalisation, Radiobiologie et Cancer, Orsay, France*

^9^ *Université Paris-Saclay, CNRS UMR, Inserm, Signalisation, Radiobiologie et Cancer, Orsay, France*

**Author Contributions Statement**

In the Author Contributions statement in the published article, there was an error; author Alberto Alonso was erroneously excluded.

This paragraph previously stated:

Conceptualization: FC, JA, MC and YP. Data curation: FC, AA, JS, MC, JA, RM, JPas, FL, BA, LC, MM, JP, JD, and YP. Formal analysis: FC, JS, and JP. Investigation: FC, AA, MM, RM, BA, DP, DA, and YP. Methodology: JS, AA, DP, DA, and YP. Project administration: FC. Supervision: FC, JA, and AA. Writing-original draft: FC, AA, JS, and YP. Writing-review and editing: FC, YP, JA, JS, RM, JP, JPas, LC, DP, DA, and AA. All authors contributed to the article and approved the submitted version.

The corrected paragraph appears below:

Conceptualization: FC, JA, MC, and YP. Data curation: FC, AA, JS, MC, JA, RM, JPas, FL, BA, LC, MM, JP, JD, AAlon, and YP. Formal analysis: FC, JS, and JP. Investigation: FC, AA, MM, RM, BA, DP, DA, AAlon, and YP. Methodology: JS, AA, DP, DA, and YP. Project administration: FC. Supervision: FC, JA, and AA. Writing-original draft: FC, AA, JS, and YP. Writing review and editing: FC, YP, JA, JS, RM, JP, JPas, LC, DP, DA, AAlon, and AA. All authors contributed to the article and approved the submitted version.

The authors apologize for this error and state that this does not change the scientific conclusions of the article in any way.

The original article has been updated.

